# Diabetes without Overt Cardiac Disease Is Associated with Markers of Abnormal Repolarization: A Case-Control Study

**DOI:** 10.3390/life12081173

**Published:** 2022-07-31

**Authors:** Tomer Stahi, Keren Kaminer, Itay Shavit, Udi Nussinovitch

**Affiliations:** 1 Sackler Faculty of Medicine, Tel Aviv University, Tel Aviv 6329302, Israel; tomer1231@gmail.com (T.S.); itayshavitmail@gmail.com (I.S.); 2 Department of Endocrinology, Rabin Medical Center, Petach Tikva 4941492, Israel; kerenkaminer@gmail.com; 3 Alpha Helix Ventures, Petach Tikva 4921352, Israel

**Keywords:** diabetes mellitus (DM), diabetic cardiomyopathy (DbCM), total cosine R to T (TCRT)

## Abstract

Patients with diabetes mellitus (DM) are prone to advanced atherosclerosis, microvascular disease, and tissue fibrosis. Despite the increased risk for arrhythmias, little is known about cardiac repolarization abnormalities in DM. We aimed to determine whether abnormal T-wave morphology markers are common among patients with DM and no known cardiac disease. Patients were recruited and classified as having DM or impaired fasting glucose (IFG) according to accepted guidelines. Total cosine R to T (TCRT) and T-wave morphology dispersion (TMD) were computed with custom-designed software for randomly selected and averaged beats. Among 124 patients recruited; 47 were diagnosed with DM and 3 IFG. DM patients and the control group had similar clinical characteristics, other than statins and anti-diabetic drugs, which were more common among DM patients. Patients with DM/IFG had decreased TCRT values computed from a random beat (0.06 ± 0.10 vs. 0.43 ± 0.07, *p* < 0.01) and an average beat (0.08 ± 0.09 vs. 0.44 ± 0.06, *p* < 0.01), when compared with the control group. TMD parameters did not differ. In conclusion, TCRT is reduced in patients with DM and no known cardiac diseases. Further research is required to investigate whether repolarization-associated changes in DM are the consequence of subclinical atherosclerosis, diabetic cardiomyopathy, or a combination of the two.

## 1. Introduction

According to some estimates, 382 million people worldwide have diabetes mellitus (DM), and this number is expected to reach 592 million by the year 2035 [1]. Atherosclerotic cardiovascular disease remains the main cause of heart failure among patients with DM, attributing to approximately two-thirds of deaths in these patients. During the last three decades, rates of cardiovascular diseases in persons with DM decreased, but they remained significantly higher than those observed in persons without DM [2]. Other than microvascular and macrovascular heart disease, patients with DM are often affected with cardiac autonomic neuropathy (CAN) [3], which may compromise ventricle function and increase the risk of arrhythmias and death [4]. Cardiomyopathy may also develop in patients with DM due to processes other than myocardial ischemia. Accordingly, diabetic cardiomyopathy (DbCM) is diagnosed in the absence of coronary atherosclerosis, valvular disease, and hypertension [5]. DbCM evolves as a consequence of a myriad of mechanisms, including glucose and lipid toxicity, inflammation imbalances, increased myocardial fibrosis, CAN, and microvascular changes [4].

Ventricular arrhythmias are commonly found in patients with DM. They may emerge as a result of myocardial fibrosis (a recognized substrate for reentrant arrhythmias), cardiac ischemic events, and CAN [6]. Animal models of DM revealed several electrophysiological changes, including altered expression of the potassium ion channel, prolongation of the QT interval, and alterations in connexin 43 and 45 expression and phosphorylation [7], all of which may contribute to increased arrhythmic risk in DM.

Total cosine R to T (TCRT) is a novel electrocardiographic marker that reflects the spatial angle between ventricular depolarization and repolarization [8]. Negative TCRT values are associated with an increased risk of cardiac events and adverse prognosis in various patient populations [9,10]. This marker was reported to possess significant advantages in comparison to other methods of evaluating the spatial angle between the QRS complex and T-wave loop orientations [11]. T-wave morphology dispersion (TMD), a marker of variation in T-waves between individual leads, was also reported to be predictive of cardiac death [12].

There is a paucity of information on the association between TCRT, TMD, and DM. The association between TCRT and hypoglycemia has been reported in a few small cohorts, with conflicting results [13,14], and in other cohorts consisting of patients with various cardiac and non-cardiac conditions, as well as cardiovascular risk factors other than DM [15,16,17]. These reports did not directly compare two groups that differ only with regard to the presence of DM, and limited information on the association between T-wave morphology parameters and DM was provided, overall. Notably, there are no specific reports in the medical literature investigating the association between TMD and diabetes. Our aim is to bridge this gap and specifically study the degree to which DM, regardless of the presence of ischemic heart disease, is associated with abnormal repolarization, as reflected by T wave morphology parameters. We hypothesized that repolarization abnormalities will be commonly found in diabetic patients, even without a clinically overt cardiac disease. We also speculated that these markers, if indeed found, may be associated with adverse long-term outcomes.

## 2. Materials and Methods

### 2.1. Study Overview

This comparative case-control study received approval from the local institutional review boards (Meir Medical Center, #0074-18-MMC, Kfar Saba, Israel; Sheba Medical Center, #5003/07, Ramat Gan, Israel) and fulfilled the ethical guidelines of the most recent Declaration of Helsinki. All participants provided written informed consent.

### 2.2. Study Participants

Enrollment occurred from June 2008 to June 2010. Volunteers were recruited from the Executive Health Screening Program Clinic (a preventive program for early detection and treatment of health hazards, Sheba Medical Center, Ramat Gan, Israel). They provided a medical history, underwent physical examination, cardiac stress test, resting ECG, chest X-ray, complete blood count, and blood chemistry and were included based on the absence of cardiopulmonary disease. Hypertension, smoking, and dyslipidemia were not exclusion criteria. Included patients were categorized as having DM or IFG according to accepted thresholds of hemoglobin A1c (HGB-A1c), fasting glucose levels and random plasma glucose levels [18].

Patients were excluded based on age <18 years, pregnancy, history of any surgery, history of malignancy, any complaint that might be cardiac-related, especially if it resulted in additional diagnostic testing, regardless of the findings. Specifically, patients were interviewed on symptoms that might be related to undiagnosed ischemic heart disease or heart failure. Additionally, medical records were reviewed for any documentation of cardiovascular disease including past medical history of a myocardial infarction, stroke, or abnormal cardiac perfusion, structure, and function. Patients were excluded if they had undergone coronary angiography, regardless of the extent of atherosclerotic disease or a need for percutaneous intervention. Cardiac CT angiography or cardiac perfusion imaging conducted for reasons other than routine evaluation or for medical clearance to participate in physical activity were considered exclusion criteria. Additional exclusion criteria were any history of sudden cardiac death in a family member younger than age 40 years or a congenital heart disease in a first- or second-degree family member (either structural, functional, or a diagnosis of cardiac channelopathy). Patients with atrial fibrillation or a pacemaker were also excluded from the study. Notably, ischemic heart disease in a family member at an older age was not an exclusion criterion. Patients who were excluded from the cohort based on medical history did not undergo ECG or further evaluation within the context of the study.

### 2.3. Electrocardiograph Procedure

Participants were asked not to smoke, drink caffeinated beverages, or take other stimulants starting 3 h before the ECG study and to avoid strenuous exercise for 24 h prior to it. In all cases, the test was conducted between 9.00 a.m. and 12.00 p.m. Room temperature was maintained at 22–24 °C. Before starting the test, participants were asked to lay without moving for 10 min. Resting ECG measurements were taken with a designated, high-resolution commercial ECG (1200HR PC-ECG, Norav Medical, Yokne’am Illit, Israel). The subject’s skin was cleansed with alcohol prior to electrode placement to decrease noise level. Leads were positioned according to the standard 12-lead system. The patients were weighed and height was measured to compute body mass index (BMI). Blood pressure was recorded twice with an automated commercial sphygmomanometer (Welch Allyn 4200B-E1, Auburn, NY, USA) and values were averaged.

### 2.4. Repolarization Analysis

**Figure 1:**
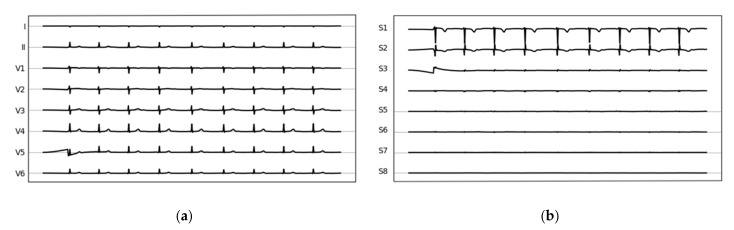
Example for an original ECG tracing (**a**) and derived singular value decomposed time-series vectors (**b**).

**Figure 2:**
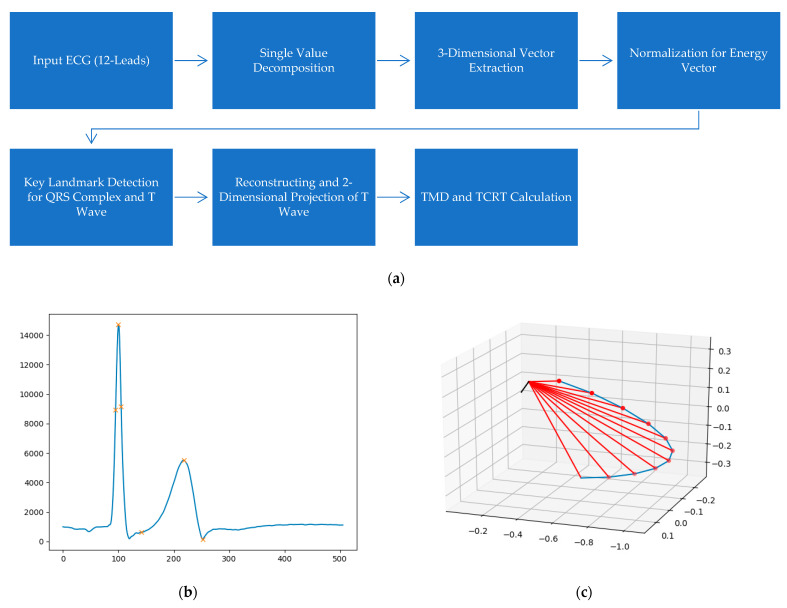
(**a**) Schematic representation of the steps used for computation of TMD and TCRT. (**b**) The $E_{3d}$ vector was used to determine R peak start, R peak, R peak end, T-wave start, T-wave peak, T-wave end; marked from left to right, respectively). R peak start and end in the presented waveform were determined in accordance with the maximum upslope and downslope, respectively [19]. (**c**) TCRT plot. T wave peak vector and QRS vectors (from R peak start to R peak end) are marked in black and red, respectively. Each angle is calculated and averaged, allowing TCRT computation.

To compute T-wave morphology parameters, the time series was exported to a binary file format. The three-dimensional vector representation of the electrical signal was accomplished by applying singular value decomposition to the eight independent surface ECG leads to produce a system of three independent orthogonal leads (e.g., $S_{3}=\left(s_{1},\;s_{2},s_{3}\right)$) that contained 99% of the ECG energy (Figure 1) [17]. Data normalization generated an “Energy vector” ($e.g.,\;\;E_{3d}={\left|\left|s_{1},\;s_{2},s_{3}\right|\right|}_{2})$ used to determine QRS and T wave landmarks (e.g., QRS peak, T wave start, peak, and end). Spatial and temporal parameters were computed based on $E_{3d}$ and $S_{3}=\left(s_{1},\;s_{2},s_{3}\right)$, according to accepted standards (Figure 2) [19]. TCRT is computed by calculating the cosine values between the 3-dimensional QRS and the T-wave loop vectors within the optimized decomposition space. The variable does not have units and negative values correspond to large differences in the rotational orientation of the two loops. TMD is a measure of differences between T-wave shapes in individual leads, calculated as the average of angles between all possible pairs of reconstructed vectors of individual ECG leads created from the T-wave loop. Small values indicate similar T-wave morphology between leads [20]. TMDpre (which describes the morphological changes from the start of the T-wave to the T-wave peak) and TMDpost (changes from T-wave peak to T-wave end) were also calculated [19]. All of the above parameters were derived from a single beat and from an averaged beat.

The repolarization analysis algorithm was written in Python 3.6 using NumPy and the external python library BIOSPPy [21]. The R-wave detection algorithm was used to separate individual beats. Measurements that contained displaced leads or high electrical interfaces despite filtering were excluded from analysis. The Python library Matplotlib was used to visualize the tracings to ensure the technical quality of the ECG data.

Further ECG analysis was automatically performed from V5 using PC-ECG 1200 Measurement software version 5.514 (Norah Medical, Yokne’am Illit, Israel) and then manually verified by a blinded investigator (IS). The end of the T wave was determined by the tangent method (e.g., intersection of the isoelectric line with the tangent to the downward slope of the T wave). The QT interval was measured from the beginning of the QRS until the end of the T wave. Corrected QT (QTc) was calculated according to Bazzet’s formula. The Tp-e interval was measured from the peak of the T wave until the end of the T wave. The Tp-e/QT and Tp-e/QTc ratios were further calculated from these measurements.

### 2.5. Statistical Analysis

Data were analyzed using JMP Pro version 16.0.0 (SAS Institute, Cary, NC, USA) and Medcalc version 19.1.5 (MedCalc Software bvba, Ostend, Belgium). Results are presented as mean and standard error of mean (SEM). Abnormal results were defined as more than 2 standard deviations from the normal range. Findings were compared between the groups using the Kruskal–Wallis one-way analysis test and Fisher’s Exact Test. A multivariate analysis was performed by including the following variables in a linear fitting model: DM/IFG, age, sex, height, weight, BMI, dyslidemia, hypertension, family history of ischemic heart disease, treatment with aspirin, angiotensin-converting-enzyme inhibitors or angiotensin II receptor blockers (ACEI/ARBs), calcium channel blockers (CCBs), beta blockers, statins, and thiazide therapy. A *p*-value less than 0.05 was considered statistically significant.

## 3. Results

Overall, 175 patients were recruited. Among them, 51 were excluded due to a diagnosis or suspicion of cardiovascular abnormalities. These included 11 with a history of myocardial infarction (MI), 16 who underwent coronary angiography not within the context of MI (of whom 5 underwent percutaneous coronary intervention), 6 who underwent coronary artery bypass graft surgery, 5 who had a family history of sudden death at a young age, 2 who were pregnant, and 11 with a history of surgical procedures or suspected or treated malignancy.

The remaining 124 volunteers were included in the study and underwent high-resolution ECG study. Among them, 47 were diagnosed with DM and 3 with IFG (40.3% of the study group). Patients’ characteristics are outlined in Table 1.

Patients with or without DM/IFG had a similar mean age, male to female ratio, height, weight, and BMI. There was a tendency toward an increased proportion of obese individuals (e.g., BMI > 30 kg/m^2^) in the DM/IFG group (36 vs. 23%, *p =* 0.11). Both groups had similar proportions of current smokers, past smokers, hypertension, family history of heart disease, as well as statistically similar rates of treatment with CCBs, beta blockers, and thiazides. There was a tendency toward an increased rate of aspirin (*p =* 0.07) and ACEI/ARBs treatment (*p =* 0.06) in the DM/IFG group. Mean systolic and diastolic blood pressure values were similar in both groups. Despite similar rates of dyslipidemia (66.2% vs. 76.0%, *p =* 1.00), patients with DM were more likely to be prescribed statins (60.0 vs. 36.5%, *p <* 0.01).

DM/IFG had been diagnosed an average of 14.1 ± 1.6 years prior to inclusion. Ten patients (20.0%) were diagnosed with type 1 DM or maturity onset diabetes of the young, and the rest had type 2 DM. Five of the patients with DM (10.0%) also had a past history of gestational DM. Mean HGB-A1c levels were 7.3 ± 0.2% at inclusion. In total, 2 patients (4.0%) were diagnosed with proteinuria, 11 (22.0%) with diabetic retinopathy, and 7 (14%) had undergone laser photocoagulation therapy. Fifteen patients (30.0%) had symptoms suggestive of diabetic neuropathy, but they did not undergo nerve conduction velocity tests prior to inclusion. A total of 33 patients (66.0%) were treated with metformin, 9 (18.0%) with sulfonylurea, 3 (6.0%) received dipeptidyl peptidase 4 (DPP-4) inhibitors, and 21 (42.0%) were treated with insulin.

**Figure 3:**
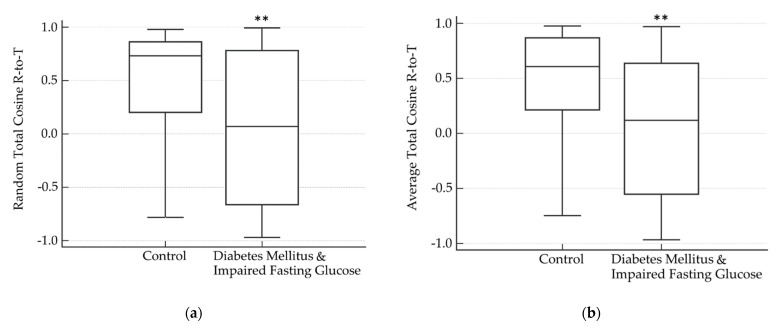
Comparison of Total Cosine R-to-T (TCRT) parameters in patients with or without diabetes mellitus or impaired fasting glucose. Lower mean values for TCRT obtained from a random beat (**a**) and an averaged beat (**b**). ** *p <* 0.01.

T-wave morphology parameters were calculated for all patients. Results are outlined in Table 1. Significantly lower TCRT values were found in patients with DM/IFG, as compared with those computed for the control group, from a random beat (0.06 ± 0.10 vs. 0.43 ± 0.07, respectively, *p <* 0.01; Figure 3a) and from an average beat (0.08 ± 0.09 vs. 0.44 ± 0.06, respectively, *p <* 0.01; Figure 3b). No difference was observed in any TMD parameter. Using a multivariate analysis of the association between possible confounders and the average TCRT, the only significant variable found to have a significant correlation with TCRT results was DM/IFG (*p =* 0.01).

In four patients (two with DM/IFD and two without DM/IFG), the recording was terminated shortly before 10 s had elapsed, thereby preventing automatic measurement of Tp-e, QT, and QTc. There was no significant difference in QT and QTc amongst the groups. Tp-e and Tp-e/QT were significantly higher in DM/IFG (69.8 ± 1.8 ms vs. 65.5 ± 1.3 ms, *p =* 0.01, and 0.19 ± 0.01 vs. 0.17 ± 0.00, *p <* 0.01, respectively), although absolute differences were small. The Tp-e/QTc ratios were also slightly higher in DM/IFG, however, the significance level was borderline (*p =* 0.054).

## 4. Discussion

There is a paucity of information on the association between TCRT and DM. Although one large cohort study that evaluated T-wave morphology parameters and outcomes included a substantial number of patients with diabetes (833/1946), the association between TCRT and DM was not reported [12]. A few small studies have investigated the association between TCRT and hypoglycemia. Chow et al. found that TCRT response in hypoglycemia was similar in 12 DM and 11 matched non-DM patients [13]. In contrast, Koivikko et al. reported that hypoglycemia was associated with a decrease in TCRT values in 16 patients with type 1 DM [14]. They also found a tendency toward lower TCRT values at rest when glucose levels were normal, but the study group was too small to enable definitive conclusions [14]. In addition, Kenttä et al. suggested that reduced recovery of TCRT-RR values following exercise were associated with diabetes [22]. Huang et al. [15] included a mixed population of patients of whom 39% had diabetes. They reported that a TCRT cutoff of −0.473 was associated with increased cardiovascular mortality. Despite the fact that DM patients were equally distributed among those who had TCRT values lower and higher than −0.473 (44 vs. 42%, respectively, *p =* 0.613), DM patients who also had TCRT values ≤−0.473 were found to have worse prognoses [15]. Friedman [16] also reported that DM is associated with decreased TCRT (−0.200 ± 0.549). Yet, the cohort included a mixed group of patients with various medical conditions, including valvular diseases and coronary heart disease, which confounded the interpretation of the results. Furthermore, the TCRT of non-diabetic patients was not reported [16]. May et al. suggested that increased frontal QRS-T angle in DM is predictive of mortality [23]. Yet, differences in classification of normal ranges of the frontal QRS-T angle was suggested to influence data interpretation [24]. In addition, this study did not compute TCRT, which is based on singular value decomposition. Notably, the TCRT parameter was designed to address the problem of wide, spatially curved QRS loops so that no single vector can reliably represent their orientation [11].

The current study is the first to investigate the association between groups with similar clinical characteristics, largely differing only in regard to the presence of DM. Furthermore, none of the included patients had a history of valvular disease or had sustained a myocardial infarction. We found that DM, regardless of other cardiovascular risk factors, was associated with significantly lower TCRT values, computed either from a randomly selected beat or from an averaged beat. Additionally, none of the TMD parameters were significantly altered in DM. On multivariate analysis, a confounder was not found amongst the investigated cardiovascular risk factors and the demographic parameters. Yet, we cannot exclude the fact that an unknown cofounder was not included in the multivariate model, which may have contributed to the present results. The absolute TCRT values found in the present study were higher than those previously reported for patients with DM [16]. Yet, incomplete data on the characteristics of patients included in other studies limit between-study comparisons. Notably, most of our patients were managed with treatments other than insulin, and they had a mean HGB-A1c of 7.3 ± 0.2%, which is within the recommended range according to the American College of Physicians (ACP) guidelines and close to the recommended target values according to the American Diabetes Association (ADA) [25]. We cannot predict what the results would have been if non-compliant patients with excessively high HGB-A1c levels were included. Our DM patient population was characterized with disease duration of 14.1 ± 1.6 years. Thus, it remains to be explored whether inclusion of patients with a longer duration or a higher degree of non-cardiac complications would have been associated with altered TCRT results. Additionally, the extent to which CAN, upon its emergence in some patients, would be associated with abnormal T-wave morphology is still unknown.

Our results are in agreement with the findings of other abnormal markers of repolarization in DM. These include prolonged Tp-e Interval, Tp-e/QT ratio, and Tp-e/QTc ratio, suggestive of increased transmural dispersion of repolarization [26]. Notable, similar findings as to the significantly higher Tp-e and Tp-e/QT ratio were also found in the present study, although, absolute differences between the groups were small. Moreover, increased dispersion of QT interval in DM may be suggestive of heterogeneous cardiac repolarization [27], although the prognostic implications of QT interval and QT dispersion measurements in DM have been challenged [28].

The main limitation of the current study is the uncertainty as to the etiology of abnormal TCRT in patients with DM. Patients did not undergo coronary angiography or coronary CT angiography. Therefore, the diagnosis of coronary atherosclerosis could not be ascertained or rejected. In addition, none of our patients received sodium-glucose cotransporter 2 (SGLT2) inhibitor, a drug that was shown to markedly improve cardiovascular outcome in patients with DM type 2 [29,30]. Interestingly, treatment with SGLT2 inhibitors was reported to be associated with a reduced Tp-e/QT ratio and QT corrected dispersion, suggestive of a relative decrease in the risk for ventricular arrhythmias [31]. We cannot predict whether results would be different if SGLT2 treatment was prescribed to some or all of our patients. Moreover, the current study was not powered to investigate the possible effects of sulfonylureas on repolarization parameters in light of the relatively small number of included patients who received this treatment. Therefore, the association between medical therapy and repolarization parameters should be further studied in future cohorts. Notably, there are no specific thresholds to define T-wave morphology parameters as abnormal, and a wide range of results were reported in healthy patients and in those with disease [8]. This may stem, at least in part, from differences in the algorithms used to calculate T-wave morphology parameters, because commercial options are not available. Furthermore, the number of DM patients who developed non-cardiac complications in this study was relatively small. Therefore, the present study cannot determine whether specific neuropathic, retinopathic, or nephropathic changes in DM are associated with more negative TCRT values. Nevertheless, the present study suggests that DM induces profound changes in repolarization parameters, and it specifically and more significantly affects TCRT. The diagnostic and the prognostic implications of changes in T wave morphology in DM should be the focus of future studies.

## 5. Conclusions

TCRT is reduced in patients with DM who have no known cardiac diseases. Further research is required to determine whether repolarization-associated changes in DM are the consequence of subclinical atherosclerosis, DbCM, CAN, or a combination of the three. Additionally, it remains to be determined whether TCRT is influenced by treatment with SGLT2 inhibitors as well as by other treatments in DM patients, and, if such changes occur, whether they correlate with improved cardiovascular outcomes.

## Figures and Tables

**Table 1:** Demographic, clinical characteristics, and T wave morphology parameters in patients with and without diabetes mellitus or impaired fasting glucose (DM/IFG).

Parameter	Non-DM/IFG (n = 74)	DM/IFG (n = 50)	*p* Value
Age (years)	57.3 ± 1.5	58.4 ± 1.8	NS
M/F	40/34	26/24	NS
Height (m)	1.69 ± 0.01	1.71 ± 0.01	NS
Weight (kg)	79.2 ± 1.9	82.1 ± 2.0	NS
BMI (kg/m^2^)	27.5 ± 0.5	28.1 ± 0.7	NS
Obesity * (%)	23.0	36.0	NS
Current smoker (%)	14.9	14.0	NS
Past smoker (%)	24.7	26.0	NS
Hypertension (%)	39.5	68.0	NS
CVD ** (%)	0	0	NS
s/p angiography (%)	0	0	NS
FHx of IHD (%)	37.8	40	NS
Dyslipidemia (%)	66.2	76.0	NS
SBP (mmHg)	130.5 ± 2.2	134.4 ± 2.6	NS
DBP (mmHg)	79.0 ± 1.1	75.5 ± 1.5	NS
Aspirin (%)	35.1	50.0	NS
ACEI/ARBs (%)	36.5	52.0	NS
CCB (%)	20.3	30.0	NS
Beta blockers (%)	29.7	34.0	NS
Thiazides (%)	16.2	26.0	NS
Statins (%)	36.5	60.0	<0.01
Random TCRT	0.43 ± 0.07	0.06 ± 0.10	<0.01
Random TMD (deg)	19.6 ± 1.9	21.5 ± 2.5	NS
Random TMDpre	18.0 ± 2.0	21.3 ± 2.7	NS
Random TMDpost	16.8 ± 2.2	19.7 ± 3.1	NS
Average TCRT	0.44 ± 0.06	0.08 ± 0.09	<0.01
Average TMD (deg)	19.7 ± 1.8	21.0 ± 2.5	NS
Average TMDpre	18.7 ± 1.8	20.9 ± 2.4	NS
Average TMDpost	16.8 ± 2.1	18.8 ± 2.9	NS
QT (ms)	380.6 ± 3.4	371.5 ± 4.8	NS
QTc (ms)	387.4 ± 2.4	393.1 ± 3.3	NS
Tp-e (ms)	65.5 ± 1.3	69.8 ± 1.8	0.01
Tp-e/QT	0.17 ± 0.00	0.19 ± 0.01	<0.01
Tp-e/QTc	0.17 ± 0.00	0.18 ± 0.00	NS

* Defined as BMI > 30 kg/m^2^, ** Previous myocardial infarction, stroke, or any symptoms suggestive of cardiovascular disease, regardless of the clinical workup conducted, or any angiographic, tomographic, or perfusion mapping suggestive of coronary atherosclerotic disease. s/p—status post, SBP—systolic blood pressure, DBP—diastolic blood pressure, FHx—family history, IHD—ischemic heart disease, ACEI—angiotensin-converting-enzyme inhibitors, ARBs—Angiotensin II receptor blockers, CCB—calcium channel blockers, TCRT—Total cosine R to T, TMD—T-wave morphology dispersion, QTc—corrected QT interval, Tp-e—T peak to T end duration.

## Data Availability

Data will be made available upon a reasonable request.

## References

[1] Guariguata L., Whiting D.R., Hambleton I., Beagley J., Linnenkamp U., Shaw J.E. (2014). Global estimates of diabetes prevalence for 2013 and projections for 2035. Diabetes Res. Clin. Pract..

[2] Low Wang C.C., Hess C.N., Hiatt W.R., Goldfine A.B. (2016). Clinical Update: Cardiovascular Disease in Diabetes Mellitus: Atherosclerotic Cardiovascular Disease and Heart Failure in Type 2 Diabetes Mellitus—Mechanisms, Management, and Clinical Considerations. Circulation.

[3] Nussinovitch U., Cohen O., Kaminer K., Ilani J., Nussinovitch N. (2012). Evaluating reliability of ultra-short ECG indices of heart rate variability in diabetes mellitus patients. J. Diabetes Complicat..

[4] Mauricio D., Alonso N., Gratacos M. (2020). Chronic Diabetes Complications: The Need to Move beyond Classical Concepts. Trends Endocrinol. Metab..

[5] Jia G., Hill M.A., Sowers J.R. (2018). Diabetic Cardiomyopathy: An Update of Mechanisms Contributing to This Clinical Entity. Circ. Res..

[6] Eren H., Kaya U., Ocal L., Ocal A.G., Genc O., Genc S., Evlice M. (2019). Presence of fragmented QRS may be associated with complex ventricular arrhythmias in patients with type-2 diabetes mellitus. Acta Cardiol..

[7] Grisanti L.A. (2018). Diabetes and Arrhythmias: Pathophysiology, Mechanisms and Therapeutic Outcomes. Front. Physiol..

[8] Nussinovitch U. (2020). Normal ranges and potential modifiers of T-wave morphology parameters among healthy individuals: A meta-analysis. Pacing Clin. Electrophysiol..

[9] Batchvarov V., Hnatkova K., Ghuran A., Poloniecki J., Camm A.J., Malik M. (2003). Ventricular gradient as a risk factor in survivors of acute myocardial infarction. Pace.

[10] Tse G., Gong M., Wong C.W., Chan C., Georgopoulos S., Chan Y.S., Yan B.P., Li G., Whittaker P., Ciobanu A. (2018). Total cosine R-to-T for predicting ventricular arrhythmic and mortality outcomes: A systematic review and meta-analysis. Ann. Noninvasive Electrocardiol..

[11] Hnatkova K., Seegers J., Barthel P., Novotny T., Smetana P., Zabel M., Schmidt G., Malik M. (2018). Clinical value of different QRS-T angle expressions. Europace.

[12] Pirkola J.M., Konttinen M., Kentta T.V., Holmstrom L.T.A., Junttila M.J., Ukkola O.H., Huikuri H.V., Perkiomaki J.S. (2018). Prognostic value of T-wave morphology parameters in coronary artery disease in current treatment era. Ann. Noninvasive Electrocardiol..

[13] Chow E., Bernjak A., Walkinshaw E., Lubina-Solomon A., Freeman J., Macdonald I.A., Sheridan P.J., Heller S.R. (2017). Cardiac Autonomic Regulation and Repolarization During Acute Experimental Hypoglycemia in Type 2 Diabetes. Diabetes.

[14] Koivikko M.L., Karsikas M., Salmela P.I., Tapanainen J.S., Ruokonen A., Seppanen T., Huikuri H.V., Perkiomaki J.S. (2008). Effects of controlled hypoglycaemia on cardiac repolarisation in patients with type 1 diabetes. Diabetologia.

[15] Huang H.C., Lin L.Y., Yu H.Y., Ho Y.L. (2009). Risk stratification by T-wave morphology for cardiovascular mortality in patients with systolic heart failure. Europace.

[16] Friedman H.S. (2007). Determinants of the total cosine of the spatial angle between the QRS complex and the T wave (TCRT): Implications for distinguishing primary from secondary T-wave abnormalities. J. Electrocardiol..

[17] Poulikakos D., Hnatkova K., Banerjee D., Malik M. (2018). Association of QRS-T angle and heart rate variability with major cardiac events and mortality in hemodialysis patients. Ann. Noninvasive Electrocardiol..

[18] Pippitt K., Li M., Gurgle H.E. (2016). Diabetes Mellitus: Screening and Diagnosis. Am. Fam. Physician.

[19] Acar B., Yi G., Hnatkova K., Malik M. (1999). Spatial, temporal and wavefront direction characteristics of 12-lead T-wave morphology. Med. Biol. Eng. Comput..

[20] Nayyar S., Hasan M.A., Roberts-Thomson K.C., Sullivan T., Baumert M. (2017). Effect of Loss of Heart Rate Variability on T-Wave Heterogeneity and QT Variability in Heart Failure Patients: Implications in Ventricular Arrhythmogenesis. Cardiovasc. Eng. Technol..

[21] https://github.com/PIA-Group/BioSPPy.

[22] Kentta T., Karsikas M., Junttila M.J., Perkiomaki J.S., Seppanen T., Kiviniemi A., Nieminen T., Lehtimaki T., Nikus K., Lehtinen R. (2011). QRS-T morphology measured from exercise electrocardiogram as a predictor of cardiac mortality. Europace.

[23] May O., Graversen C.B., Johansen M.O., Arildsen H. (2018). The prognostic value of the frontal QRS-T angle is comparable to cardiovascular autonomic neuropathy regarding long-term mortality in people with diabetes—A population based study. Diabetes Res. Clin. Pract..

[24] Tanriverdi Z., Besli F., Gungoren F., Tascanov M.B. (2020). What is the normal range of the frontal QRS-T angle?. Diabetes Res. Clin. Pract..

[25] Smetana G.W., Nathan D.M., Dugdale D.C., Burns R.B. (2019). To What Target Hemoglobin A1c Level Would You Treat This Patient With Type 2 Diabetes?: Grand Rounds Discussion From Beth Israel Deaconess Medical Center. Ann. Intern. Med..

[26] Tokatli A., Kilicaslan F., Alis M., Yiginer O., Uzun M. (2016). Prolonged Tp-e Interval, Tp-e/QT Ratio and Tp-e/QTc Ratio in Patients with Type 2 Diabetes Mellitus. Endocrinol. Metab..

[27] Kittnar O. (2015). Electrocardiographic changes in diabetes mellitus. Physiol. Res..

[28] Tokatli A., Yiginer O., Kilicaslan F., Uzun M. (2015). Effect of cardiac rehabilitation on ventricular repolarization in patients with type 2 diabetes and coronary heart disease: Non-invasive quantification via transmural dispersion of repolarization. Heart Lung.

[29] Zinman B., Wanner C., Lachin J.M., Fitchett D., Bluhmki E., Hantel S., Mattheus M., Devins T., Johansen O.E., Woerle H.J. (2015). Empagliflozin, Cardiovascular Outcomes, and Mortality in Type 2 Diabetes. N. Engl. J. Med..

[30] Wiviott S.D., Raz I., Bonaca M.P., Mosenzon O., Kato E.T., Cahn A., Silverman M.G., Zelniker T.A., Kuder J.F., Murphy S.A. (2019). Dapagliflozin and Cardiovascular Outcomes in Type 2 Diabetes. N. Engl. J. Med..

[31] Sato T., Miki T., Furukawa S., Matsuura B., Hiasa Y., Ohnishi H., Tanno M., Miura T. (2019). Longitudinal impact of dapagliflozin treatment on ventricular repolarization heterogeneity in patients with type 2 diabetes. J. Diabetes Investig..

